# The Functions and Unique Features of LncRNAs in Cancer Development and Tumorigenesis

**DOI:** 10.3390/ijms22020632

**Published:** 2021-01-10

**Authors:** Kenzui Taniue, Nobuyoshi Akimitsu

**Affiliations:** 1 Isotope Science Center, The University of Tokyo, 2-11-16, Yayoi, Bunkyo-ku, Tokyo 113-0032, Japan; 2 Cancer Genomics and Precision Medicine, Division of Gastroenterology and Hematology-Oncology, Department of Medicine, Asahikawa Medical University, 2-1 Midorigaoka Higashi, Asahikawa 078-8510, Hokkaido, Japan

**Keywords:** long noncoding RNAs (lncRNAs), subcellular localization, lncRNA-protein interactions, dysregulation of lncRNA expression, oncogenic lncRNA, tumor suppressive lncRNAs, lncRNAs with metastatic potential

## Abstract

Over the past decades, research on cancer biology has focused on the involvement of protein-coding genes in cancer development. Long noncoding RNAs (lncRNAs), which are transcripts longer than 200 nucleotides that lack protein-coding potential, are an important class of RNA molecules that are involved in a variety of biological functions. Although the functions of a majority of lncRNAs have yet to be clarified, some lncRNAs have been shown to be associated with human diseases such as cancer. LncRNAs have been shown to contribute to many important cancer phenotypes through their interactions with other cellular macromolecules including DNA, protein and RNA. Here we describe the literature regarding the biogenesis and features of lncRNAs. We also present an overview of the current knowledge regarding the roles of lncRNAs in cancer from the view of various aspects of cellular homeostasis, including proliferation, survival, migration and genomic stability. Furthermore, we discuss the methodologies used to identify the function of lncRNAs in cancer development and tumorigenesis. Better understanding of the molecular mechanisms involving lncRNA functions in cancer is critical for the development of diagnostic and therapeutic strategies against tumorigenesis.

## 1. Introduction

The central dogma that ‘DNA makes RNA, and RNA makes protein’ [1,2] has proposed that genetic information is stored in protein-coding genes, which are the conventional targets in cancer biology [3,4]. Multiple studies have shown that somatic mutations in protein-coding genes are the driving force of cancer development [5,6]. For several decades, most of the non-protein-coding region of the human genome has been considered junk DNA [7]. However, over the past decade, massive parallel sequencing technology has revealed that most the human genome is dynamically and differentially transcribed into noncoding RNAs (ncRNAs); this includes long ncRNAs (lncRNAs) [3,8,9]. LncRNAs are defined as RNAs larger than 200 bp that appear to have little or no coding potential [3,10,11,12]. Recent evidence from numerous studies has suggested that the complex processes regulating cancer development are not only controlled by protein-coding RNAs but are also regulated by the noncoding portions of the genome [13].

RNA has a diverse set of roles and is more than just an intermediate molecule between genes and proteins [14]. After the discovery of messenger RNA, many other RNAs were identified, such as small nuclear RNAs that are involved in splicing regulation [15], small nucleolar RNAs involved in ribosome biogenesis [16], and ribosomal RNAs and transfer RNAs involved in translation [17]. The RNA world has become more complex with the identification of lncRNAs, which are similar to mRNA in length and splicing structure but do not encode proteins. The lncRNA *H19* was discovered as an RNA that is induced during the development of liver in mouse [18] and the lncRNA *XIST*, which is required for X inactivation, was found to be expressed exclusively from the inactive X chromosome [19]. Over the past several decades, both large and small RNAs were discovered at an unprecedented rate because of large-scale genomic projects, including the Encyclopedia of DNA Elements (ENCODE) [8,20,21,22,23,24,25,26,27,28]. However, the functional importance of most of these RNAs has remained unclear. 

Accumulating evidence indicates that lncRNAs play critical roles in diverse biological processes, including proliferation, differentiation, embryogenesis, neurogenesis, stem cell pluripotency, pathogenic infection and tumorigenesis [7,9,12,29,30,31,32,33,34,35,36,37,38,39,40]. LncRNAs also function in chromatin and genomic structural remodeling, RNA stabilization and transcriptional regulation [36,41]. Other studies reported that lncRNAs can regulate protein stability by preventing post-translational modifications associated with protein degradation [40]. In cancer, lncRNAs have tissue- and cancer type–specific expression and are expressed in correlation with several gene sets that influence the cell cycle, survival and metastasis, among other functions, which contribute to the phenotype of cancer cells [42]. Moreover, multiple lncRNAs are transcriptionally regulated by oncoproteins or tumor suppressors, which is important for cancer tumorigenesis [29,43].

In this review, we will describe the biogenesis and features of lncRNAs produced from intergenic loci and the current knowledge regarding the roles of lncRNAs in cancer. We also present an overview of the methodologies to identify the function of lncRNAs in cancer development and tumorigenesis as well as the challenges of studying them and the potential to target lncRNAs for diagnostic and therapeutic applications.

## 2. Definition of LncRNAs

Results from tiling microarrays and massive parallel sequencing technologies targeting whole genomes and transcriptomes have revealed that only <1% of the human genome encodes proteins and most of the genome is actively transcribed into ncRNAs [8]. Transcripts from the noncoding regions of the genome can be categorized as housekeeping ncRNAs or regulatory ncRNAs. Housekeeping ncRNAs, such as ribosomal RNAs (rRNAs, components of ribosomes), transfer RNAs (tRNAs, which serve as the physical link between mRNAs and the amino acids in proteins), small nuclear RNAs, (snRNAs, which are found within the splicing machinery) and small nucleolar RNAs (snoRNAs, which guide chemical modifications of other RNAs), are constitutively expressed independent of tissue and developmental stage. Regulatory ncRNAs, which are functional RNAs, include microRNAs, small interfering RNAs, Piwi-associated RNAs [44,45]. However, most regulatory ncRNAs are related to lncRNAs [46]. Several databases that provide expression, annotation, gene locus and other information on mammalian lncRNAs have recently become available [47,48,49,50,51,52,53,54,55,56,57,58,59,60].

**Figure 1:**
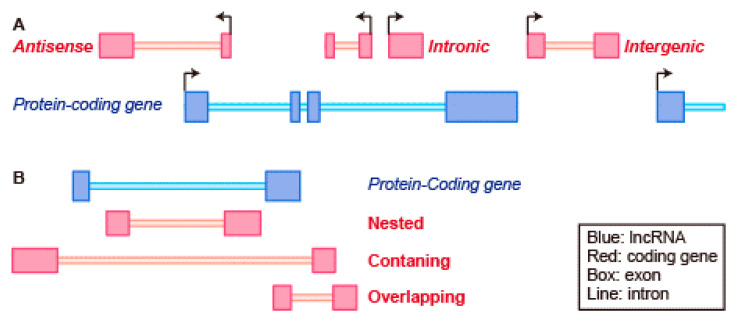
Genomic organization of coding and non-coding transcripts. (**A**) Schematic representation of the types of long non-coding transcripts (pink) that are associated with coding genes (blue). Antisense lncRNAs are transcribed from the opposite strand of protein-coding genes with which they share sequence complementarity. Intronic lncRNAs contain within protein-coding loci partly or completely. Intergenic lncRNAs do not intersect with any protein-coding genes. (**B**) Intronic lncRNA subtypes by their relationship with respect to the nearest protein-coding gene. Nested lncRNAs contained entirely within protein-coding transcripts. Containing lncRNAs are contained Protein-coding transcripts completely. Overlapped lncRNAs are neither ‘nested’ nor ‘containing’. Dark-colored box, exon; Light-colored bar, intron.

LncRNAs are located in intergenic regions, which are the genomic regions between two genes, or clustered with protein-coding genes. LncRNAs are transcribed bidirectionally or in sense or antisense directions to protein-coding genes or in intronic regions of protein-coding genes [36,46,61] (Figure 1A). When lncRNAs are expressed from intronic regions, they overlap exons of another transcript on the same or opposite strand with a ‘nested pattern,’ in which lncRNA genes are contained entirely within protein-coding transcripts; a ‘containing pattern,’ in which protein-coding transcripts are contained entirely within lncRNAs; or an ‘overlapping pattern,’ in which the lncRNA gene is neither ‘nested’ nor ‘containing’ [36] (Figure 1B). Most intronic lncRNAs have been proposed to be pre-mRNA fragments [62]. In contrast, intergenic lncRNAs remain a complete mystery. Approximately half of the intergenic lncRNAs are transcribed at 10 kb or less from protein-coding genes [63] and most can act in cis to regulate the expression of nearby genes. In contrast, the lncRNAs transcribed far from protein-coding loci have little possibility to act in cis-regulation of transcription. However, these lncRNAs can play a role in trans by association with proteins and subsequent formation of large ribonucleoprotein complexes.

## 3. LncRNA Biogenesis as well as Transcriptional and Post-Transcriptional Regulation of LncRNAs

RNA polymerase II (Pol II) transcribes more than 150,000 pre-mRNAs in the human genome, and these pre-mRNAs undergo 5′ capping, splicing, 3′ cleavage and poly-adenylation [64]. Most, but not all, lncRNAs are generated by the same transcriptional machinery as other mRNAs and are transcribed by RNA Pol II with histone modifications related to transcription initiation and elongation [9,42,61]. A set of transcribed lncRNAs, which are often spliced, are capped with 5′ terminal methylguanosine and polyadenylated at their 5′ and 3′ ends [61]. LncRNAs undergo RNA processing features like mRNA; however, whereas mRNA processing is more robustly coordinated with the transcription, splicing and polyadenylation machinery, lncRNAs are more often cleaved and prematurely terminated when transcribed. Recent studies using mammalian native elongating transcript sequencing, which can investigate genome-wide Pol II density by detecting the phosphorylation status of the Pol II C-terminal domain, revealed that Pol II pauses inefficiently at lncRNA promoters and throughout lncRNA loci, resulting in more frequent transcription termination than observed in protein-coding genes [65]. LncRNAs are also generated by several alternate pathways. RNA polymerase III transcribes non-polyadenylated lncRNAs [66,67] and some lncRNAs are excised during splicing and snoRNA production [68]. LncRNA transcripts also have fewer and shorter exons and are expressed at lower level than mRNAs [69].

Similar to protein-coding genes, lncRNA genes are globally enriched at their promoter regions with histone modifications such as H3K27ac, H3K4me3 and H3K9ac [70]. In addition, most lncRNA genes are characterized by H3K4me3 at the transcription start site and H3K36me3 along the gene body [10,42,71,72]. LncRNAs can also regulate the expression of their own genes and/or their target genes by association with chromatin-related complexes, such as the PRC2 complex, to regulate the chromatin state and their expression [10,73,74,75].

Many lncRNAs are expressed in a developmentally regulated- and cell type-dependent manner, and the expression of lncRNAs is tissue-specific compared with that of mRNAs [76]. The tissue specificity of lncRNA expression suggests that they modulate expression of specific genes through physical proximity, promoting establishment and maintenance of tissue in which function of mRNAs, other non-coding RNAs or lncRNAs linked to each other [10,11,36,46]. 

No defining biological features can be exclusively attribute to lncRNAs, because they have a generation process in common with mRNAs. Experimental support against the categorization of a lncRNA can be presented by ribosomal profiling with deep sequence technology or peptide fragments from mass spectrometry analysis that indicate translational regulation of RNAs [77,78]. However, the protein-encoding capacity does not necessarily interfere with other functions of RNA, and indeed several studies have provided evidence for mRNAs that function as not only protein-encoding molecules but also as lncRNAs [79]. Moreover a lack of function, including the lack of an extended open reading frame, provides logical evidence that many transcripts function as an RNA [80].

## 4. Features of LncRNAs

### 4.1. Evolutionary Conservation of LncRNAs

NONCODE database, which is an integrated knowledge database dedicated to ncRNAs (excluding tRNAs and rRNAs), are databases that contain over 100,000 lncRNAs, and most lncRNAs are yet to be annotated [57]. Elucidation of conservation patterns within genes and between ncRNAs and their interaction partners has greatly advanced the understanding of other ncRNAs [81,82,83]. In addition, analyzing the evolutionary conservation of lncRNAs might allow for the identification of elements and structures important for lncRNA function. LncRNAs have a greater conservation than ancient repeat sequences but less than protein-coding genes. Moreover, compared with protein-coding RNAs and other ncRNAs, lncRNAs have fewer invertebrate orthologues, except for the telomeric repeat-containing RNA (*Terra*), and have undergone rapid evolution [11,84]. Some lncRNAs are functionally conserved across species [11,85,86,87,88]. Less than 6% of zebrafish lncRNAs show sequence conservation with mammalian lncRNAs [89]. Moreover, exon regions of lncRNAs tend to be more conserved than the intergenic regions, but are significantly less conserved than mRNA exons [27,42,63,73,89].

### 4.2. Subcellular Localization and Secondary Structure of LncRNA

The subcellular localization of lncRNAs is as diverse as that observed with protein-coding mRNAs [46]. mRNAs are mainly localized to the cytoplasm where they undergo translation, whereas lncRNAs are more often located in the nucleus than in the cytoplasm [29,39,40,90,91,92,93]. However, several lncRNAs are found mostly in the cytoplasm [94,95]. The factors or sequence elements that define the specific localization of lncRNAs within either the cytoplasm or nucleus remain largely unknown.

Secondary and higher order structures are an important feature for most lncRNAs and confer thermodynamic stability [11,61,96]. RNA form hydrogen bonds on the Hoogsteen and ribose face as well as the Watson-Crick face [61]. These interactions lead to the formation of RNA secondary structures, including double helices, hairpin loops, bulges and pseudoknots, forming tertiary and higher order structures by non-Watson-Crick base-pairing [97]. The secondary structure of lncRNAs determines their function and several lncRNAs are highly conserved at the secondary structure level [36]. For example, the lncRNA *MEG3* exerts its tumor suppressor function through motifs within its secondary structure rather than its primary sequence [98]. Metastasis associated in lung adenocarcinoma transcript (*MALAT*1), which is an abundant and highly conserved lncRNA across mammals, is stabilized by forming a triple helix structure at its 3′ end [99]. Terminal differentiation-induced noncoding RNA (*TINCR*) is conserved at its 5′ end across vertebrates other than mice [93], while the 3′ end shows differences in sequence across vertebrates [36].

## 5. Roles of LncRNAs in Cancer

### 5.1. LncRNAs with Oncogenic Potential

HOX antisense intergenic RNA (*HOTAIR*), one of the most well studied lncRNAs, is a 2.2-kb antisense lncRNA that is embedded in the *HOXC* locus. *HOTAIR* was first described as interacting with EZH2 and SUZ12, members of the PRC2 complex that methylates histone H3 on lysine 27 [23,100]. *HOTAIR* interacts with LSD1, which is a demethylase that mediates enzymatic demethylation of H3K4me2 [101]. *HOTAIR* expression is correlated with poor outcome in primary breast, colon and lung tumors; this lncRNA serves as a diagnostic and prognostic marker and is considered a potential therapeutic target in different cancer types [100,102]. Noncoding RNA activated by DNA damage (*NORAD*) is induced by the DNA damage response and plays a critical role in chromosomal instability [103]. *NORAD* maintains genomic stability by sequestering PUMILIO proteins, which are evolutionally conserved RNA-binding proteins that regulate the stability and translation of mRNAs [104,105]. *NORAD* contributes to cancer development, and its expression is upregulated and associated with poor prognosis in various types of cancers, including colorectal cancer, pancreatic cancer, breast cancer, esophageal squamous cell carcinoma and bladder cancer [106,107,108,109,110,111,112]. The survival associated mitochondrial melanoma specific oncogenic non-coding RNA (*SAMMSON*), which is located 30 kb downstream of the *MITF* gene, regulates the survival of melanoma and is co-amplified with MITF in approximately 10% of human melanomas [113]. *SAMMSON* interacts with p32, a master regulator of mitochondrial homeostasis and metabolism, to increase its mitochondrial localization and pro-oncogenic function [113]. *SAMMSON* promotes an increase in rRNA maturation and protein translation in the cytosol and mitochondria via sequestration of the nuclear RNA-binding protein CARF in the cytoplasm [114]. 

Several lncRNAs, such as plasmacytoma variant translocation 1 (*PVT*1), colon cancer-associated transcripts 1 and 2 (*CCAT*1 and *CCAT*2, respectively), prostate cancer associated transcript 1 (*PCAT*1) and MYC-induced long non-coding RNA (*MINCR*), regulate the expression of the proto-oncogene *MYC*, which is located in the 8q24 locus, the most frequently amplified region in human cancers [115,116,117,118,119,120,121,122]. These lncRNAs also map in 8q24 close to the *MYC* gene [115,116,117,118,119,120,121,122]. *PVT*1 is often involved in DNA rearrangements in Burkitt’s lymphoma, multiple myeloma and gastric cancer [116,120,123,124,125,126]. *PVT*1 and MYC are co-amplified in a variety of human tumors, and the co-amplification results in MYC stabilization and proliferation of cancer cells [102,116,123]. *PVT*1 is not only a MYC target gene, but part of the same ribonucleoprotein complex together with MYC; however, *PVT*1 also contributes to tumorigenesis independent of MYC [127]. *PVT*1 depletion can induce apoptotic cell death of cancer cells [120], whereas MYC silencing does not affect cell death, suggesting different mechanisms of *PVT*1 and MYC function in different cancers [128,129]. *CCAT*1 and *CCAT*2, which map upstream of the *MYC* locus by approximately 515 kb and 333 kb in 8q24, respectively, regulate the function of MYC [118,130]. *CCAT*1 knockdown was shown to reduce the proliferation of cancer cells through cell cycle arrest and tumorigenesis of colon cancer cells in a xenograft model [119]. Conversely, over-expression of *CCAT*2, a target of Wnt signaling, increased the expression of MYC and promoted invasive tumor growth in a xenograft model [118]. *PCAT*1, a lncRNA that maps approximately 710 kb upstream of the *MYC* gene, is responsible for the post-transcriptional regulation of MYC in prostate cancer [121]. *MINCR* contributes to the regulation of Myc target genes that are involved in cell cycle and functions in cell cycle progression in Burkitt lymphoma cells [122]. Moreover, *MINCR* knockdown induces cell cycle arrest and apoptosis by reducing the expression of Myc and its downstream effectors, including cyclin A, cyclin D, CDK2 and Bcl-2, in non-small cell lung cancer cells [131]. 

### 5.2. LncRNAs with Tumor Suppressor Functions

Multiple lncRNAs have been found to play an important role in modulating tumor suppressor functions. For example, several lncRNAs regulate the expression of key tumor suppressors from the *CDKN2A/CDKN2B* locus, which encodes *p15 INK4b*, *p16 INK4a*, and *p14 ARF* genes [9,13,102,132]. The antisense noncoding transcript *p15-AS* is also transcribed from the *CDKN2A/CDKN2B* locus; *p15-AS* expression is associated with low p15 INK4b expression in leukemic cells and it represses p15 INK4b expression through modulating heterochromatin formation [133]. TCF21 antisense RNA inducing demethylation (*TARID*), an antisense RNA of TCF21 that is a tumor suppressor, binds and recruits GADD45a to the *TCF21* promoter to facilitate demethylation in several cancers, including non-small cell lung cancer, head and neck squamous cell carcinomas and ovarian cancers [134]. Growth arrest-specific transcript 5 (*GAS*5), a lncRNA that functions in embryogenesis, controls apoptosis and is downregulated in cancer [135,136,137,138]. The expression of *GAS*5 is inversely correlated with tumor size, staging, and metastasis in several tumor types, including breast, bladder, colon, pancreas, and prostate cancer [139,140].

PTEN acts as a tumor suppressor by regulating PI3K and downstream effectors such as AKT, and the expression levels of PTEN are tightly regulated [141,142]. PTEN pseudogene (*PTENP*1) is homologous to the coding sequence and a portion of the 3’ UTR of *PTEN* mRNA [143]. *PTENP*1 functions as a decoy for PTEN-targeting miR-19b and miR-20a, and its locus is selectively deleted in sporadic colon cancer and prostate cancer [143,144].

The most studied protein in the cancer research field is the tumor suppressor p53, which is called the “guardian of the genome” [145]. Understanding the mechanism of p53 tumor suppressive pathways that are regulated by lncRNAs has been progressed. Research on lncRNAs that are involved in the p53-mediated DNA damage response pathway is greatly advanced. *LincRNA-p21* is an antisense transcript of *CDKN1A*, the gene that encodes the p21 tumor suppressor, and is induced by DNA damage in a p53-dependent manner [43,146]. *LincRNA-p21* causes disruption of the G1/S cell cycle checkpoint in a p21-dependent manner through its interaction with hnRNPK to regulate *CDKN1A* expression [43,146]. The expression of *lincRNA-p21* correlates with tumor stage and invasive phenotype in colon cancer, and *lincRNA-p21* enhances sensitivity to radiation through the Wnt/β-catenin signaling pathway [147,148]. Moreover, *lincRNA-p21* causes a significant transcriptional decrease of *CTNNB1* and *JUNB* genes by its interaction with HuR in breast cancer cells [94] and impairs cell proliferation in diffuse large B cell lymphoma cell lines [149]. P21 associated ncRNA DNA damage activated (*PANDA*), an antisense RNA to *CDKN1A*, is induced during the DNA damage response in a p53-dependent manner [150]. *PANDA* interacts with the transcription factor NF-YA to repress the expression of pro-apoptotic genes, resulting in inhibition of DNA damage–induced apoptosis [150]. The lncRNA *GUARDIN* is also regulated by p53 under the DNA damage response, promoting cell survival and maintaining genome stability [151]. *GUARDIN* is transcribed from the promoter region of miR-34a, which is a p53 target gene, and acts as an RNA scaffold for the heterodimerization of BRCA1 and BRCA1-associated RING domain protein 1 (BARD1), resulting in the stabilization of BRCA1 [151]. LncRNA activator of enhancer domains (*LED*), which is induced by p53, interacts with and activates strong enhancers, including an enhancer region within the *CDKN1A* gene, to regulate cell cycle arrest following p53 activation [152]. *LED* expression is largely silenced by DNA methylation in p53 wild-type primary human acute lymphocytic leukemia as well as breast, liver and prostate tumors [152]. In addition, other lncRNAs, such as *MEG3*, which is one of the imprinted genes mapping at 14q32.3 [153], are involved in p53 function in cancer cells without being transcriptionally regulated by p53. *MEG3* shows tumor suppressor activity by activating p53 and its target genes in various cancer cell lines [154,155,156,157]. Consistent with its function as a tumor suppressor, *MEG3* expression is repressed in several tumors [154,155,156,157]. 

Activation of p53 under stress conditions stimulates the formation of paraspeckles, which are membrane-less compartments that participate in transcription and RNA processing [158]. Paraspeckle formation and maintenance depend on their interaction with the lncRNA nuclear enriched abundant transcript 1 (*NEAT*1), which has two isoforms, a 3.7 kb (*NEAT-1_1*) and a 23 kb (*NEAT-1_2*) long isoform, that are widely expressed in several tissues [159,160]. *NEAT*1 is a direct target gene of p53 [158]. *NEAT*1 expression is regulated by several types of stimuli, including influenza virus and herpes simplex virus infection, bacterial infection, LPS induction and inflammasome [161,162,163,164,165]. *NEAT*1 is overexpressed in breast cancer and acute myeloid leukemia and may play an important role in the DNA damage pathway [158,166]. Depletion of *NEAT*1 inhibited cell growth, viability and morphology of breast cancer and Burkitt’s lymphoma cells [166,167]. Moreover, the loss of *Neat1* confers resistance to chemically induced skin cancer formation in mice and promotes pancreatic transformation and cancer initiation in Kras G12D–expressing mice [158,168]. 

### 5.3. LncRNAs with Metastatic Potential

Metastasis associated in lung adenocarcinoma transcript (*MALAT*1) was identified early as a prognostic factor for lung cancer survival [169,170,171]. *MALAT*1 expression has been reported to be associated with several types of tumors, including liver, breast and colon cancer [172]. Loss-of-function studies of *MALAT*1 in mice have revealed that it is non-essential for normal tissue homeostasis during development [173,174], but depletion of *MALAT*1 in lung cancer cells leads to a significant decrease in cell motility [175]. *MALAT*1, which is localized to nuclear speckles and regulated by RNA decay machinery [176,177], regulates selective splicing and epigenetic mechanisms [99,178,179]. However, the role of *MALAT*1 in cancer metastasis is not yet fully understood.

In addition to *MALAT*1, several other cancer-associated lncRNAs are involved in the regulation of invasion, metastasis and epithelial to mesenchymal transition (EMT) of cancer cells [9,13]. *HOTAIR*, which is deregulated in several cancer types as described above [180], induces metastasis by forming a macromolecular complex with PRC2 that silences specific gene loci [100,101,181]. LncRNA-activated by TGF-β (*lncRNA-ATB*), which promotes EMT and metastasis, is induced by TGFβ signaling in hepatocellular carcinoma cells [182]. *LncRNA-ATB* competitively binds to miR-200 and activates the expression of ZEB1 and ZEB2 during EMT, whereas it promotes metastasis by interacting with interleukin-11 mRNA and enhancing Stat3 signaling [182]. Second chromosome locus associated with prostate-1 (*SChLAP*1), which is associated with poor prognosis and metastatic progression in prostate cancer, promotes prostate cancer invasion and metastasis by disrupting the activity of the SWI/SNF complex [183,184]. The lncRNA *BCAR4* is interacts with the transcription factors SNIP1 and PNUTS to promote the migration and metastasis of breast cancer cells [185]. 

## 6. Methodologies for the Study of LncRNAs in Cancer

### 6.1. Identification of LncRNAs Whose Expression Is Dysregulated in Cancer Cells

**Figure 2:**
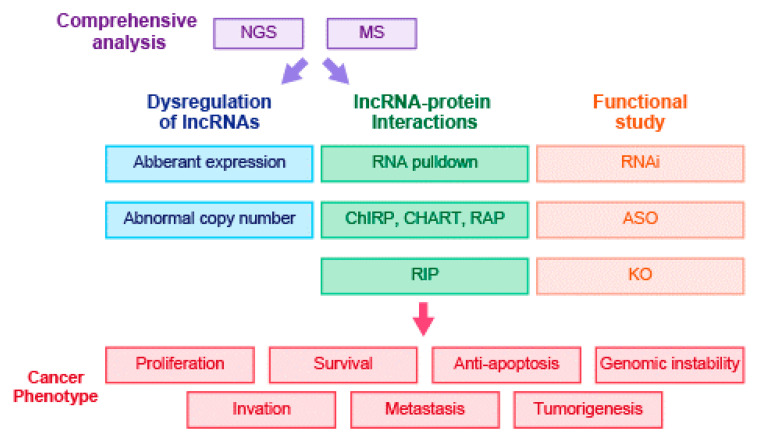
Identification and characterization of lncRNAs in cancer. Aberrant expression and alterations in genomic copy number in tumor cells lead the identification of lncRNAs related to cancer development. Subsequent in vitro and in vivo studies are used to elucidate the function of the identified lncRNAs in mediating distinct cancer phenotypes. RNA pulldown [40]; ChIRP, chromatin isolation by RNA purification [195]; CHART, capture hybridization analysis of RNA targets [196,197]; RAP, RNA antisense purification [198,199]; RNAi, RNA interference [9,29,39,40]; ASO, antisense oligonucleotide [40]; RIP, RNA immunoprecipitation [29,40]; KO, knockout [200,201,202]; NGS, next generation sequence [48,191,192,193,194]; MS, mass spectrometry [40].

The first lncRNAs that were found to be aberrantly expressed in cancer were prostate cancer antigen 3 (*PCA*3) and prostate-specific transcript 1 (*PCGEM*1), which were identified in a differential display analysis of prostate tumors and normal tissue [186,187]. Both lncRNAs are currently used as biomarkers for prostate cancer [188,189]. Before the deep-sequencing era, the technological inability to examine noncoding regions of the genome and the lack of reliable lncRNA annotation databases had prevented understanding of the big picture of the ncRNA world and the identification of functional lncRNAs. The general implementation of deep-sequencing technology solved this problem and prompted the rise of lncRNA research [9]. Recent improvements in microarray technology have greatly increased the number of probes corresponding to lncRNAs, increasing the number of lncRNAs that can be detected for aberrant expression in various types of cancer. However, with the advent of RNA-seq technology, which has a lower cost, more accurate and higher sensitivity compared with microarray technology, it is now possible to evaluate an increasingly large number of tumor samples and identify a large number of aberrantly expressed lncRNAs [190] (Figure 2). For example, using RNA-seq technology, we discovered that UHRF1 protein associated transcript (*UPAT*) expression is correlated with colorectal tumorigenesis and antisense ncRNA in the *ANA/BTG3* locus (*ASBEL*) expression is regulated by the Wnt pathway, which is dysregulated in colorectal cancer [29,40]. 

Systematic analysis by The Cancer Genome Atlas (TCGA) project [191,192,193] and the Cancer LncRNA Census (CLC), which was introduced as a part of the ICGC/TCGA Pan-Cancer Analysis of Whole Genomes (PCAWG) Consortium [48,194], has identified lncRNAs that are dysregulated and mutated in a tumor-specific manner. Both TCGA and the CLC projects have provided useful data sets and annotation data to cancer researchers and accelerated our understanding of the molecular basis of cancer including the role of lncRNAs in cancer development. 

### 6.2. LncRNA-Protein Interactions

The precise mechanism by which ncRNAs function in biological processes remains poorly understood, and comprehensive understanding of the role of lncRNAs in cancer is still in the early stage. Unlike the function of protein-coding genes, the function of lncRNAs cannot be directly inferred from the full-length sequence. However, increasing evidence suggests that most lncRNAs function through specific interactions with other bio-macromolecules, such as proteins, DNA and other RNA molecules [3,10,11,14,36]. For example, proteins are important binding partners of lncRNAs, and complexed ribonucleoprotein (RNP) particles regulate critical biological processes, such as protein degradation, mRNA transport, chromatin modification and transcription [23,29,39,40,61,74,203,204,205,206]. These interactions are important in defining lncRNA functions; however, the identification and evaluation of lncRNA-protein complexes has presented challenges. 

The most common strategy for identifying lncRNA interacting partners is by pulldown analysis to determine proteins that associate with lncRNAs of interest [13] (Figure 2). Based on the affinity purification of designed sense RNA oligonucleotide probes specific to a specific lncRNA, the procedure efficiently allows the capture of all proteins bound to lncRNAs and identification of proteins by mass spectrometry or western blot [40]. Using this strategy, we showed that *UPAT* bound to UHRF1 to interfere with its ubiquitination and degradation and revealed that *ASBEL* bound to TCF3 to regulate the expression of the tumor suppressor ATF3 in colorectal cancer [29,40]. Moreover, several studies have successfully used pulldown assays to identify and key lncRNA-binding partners. In hepatocellular carcinoma, the association of *HOTAIR* with EZH2 leads to tumor progression and aggressiveness [207]. In non-cancer cells, the p53-inducible *lincRNA-p21* was shown to bind hnRNP-K to repress gene expression and p53-mediated apoptosis in response to DNA damage and *Apela* was found to bind hnRNPL to regulate DNA damage–induced apoptosis in mouse embryonic stem cells [43,208]. 

RNA pulldown analysis can also identify proteins that bind specific lncRNAs; however, this technique can yield many artifacts resulting from non-specific interactions with misfolded lncRNAs. Furthermore, the potential of RNA to bind proteins non-specifically or only in non-physiological in vitro conditions can make interpretation of the results difficult [13]. 

In addition to RNA pulldown, other techniques have been applied, such as chromatin isolation by RNA purification (ChIRP), capture hybridization analysis of RNA targets (CHART) and RNA antisense purification (RAP), in which short biotinylated oligonucleotides complementary to the lncRNA transcript capture the target RNAs in cells. These methods can successfully identify binding proteins as well as genomic regions for lncRNAs [195,196,197,198,199]. 

Protein-lncRNA interactions have also been identified using protein-centric methods. For example, studies using RNA immunoprecipitation (RIP) have also identified lncRNA-interacting proteins and therefore functional mechanisms. In addition to the *UPAT*-UHRF1 and *ASBEL*-TCF3 complexes mentioned above, several lncRNAs, including *PCGEM*1, *PRNCR1* and *HOTAIR* that are associated with androgen receptor in prostate cancer cells were found to bind with proteins in cancer cell lines using RIP [29,40,209,210].

### 6.3. Loss-of-Function and Gain-of-Function Strategies for LncRNA Studies

To precisely evaluate the function of lncRNAs in cancer, determining their roles in cancer cell phenotypes, including cell transformation, proliferation, cell cycle deregulation, apoptosis inhibition, migration, invasiveness and tumorigenesis, is critical. In many studies, loss-of-function experiments using RNA interference (RNAi) have been used to examine lncRNA function [9,29,39,40]. However, knockdown analysis using siRNA is difficult to apply for cancer phenotypic analyses that require longer than one week, such as in tumorigenesis assays using mice, because siRNA only provides temporary inhibition. Therefore, it is necessary to prepare cell lines in which the expression of lncRNAs is stably suppressed using shRNA experiments with viruses such as lentiviruses [29,39,40].

Other oligo-based RNA knockdown technologies have been used as an alternative to repress the expression of lncRNAs in cancer cells, such as antisense oligonucleotides (ASO) or ‘gapmers’ [40]. These oligo-based strategies involve binding of ASO to the target RNA to form a DNA-RNA hybrid, which promotes RNA cleavage by ribonuclease H [211,212,213]. However, oligo-based techniques also have several limitations, such as incomplete knockdown, unpredictable off-target effects and temporary inhibition.

Knockout strategies generated by directed targeting nucleases provide a powerful tool for elucidating lncRNA function both in vitro and in vivo [214,215,216]. In particular, genome editing using the CRISPR-Cas system or zinc finger nucleases (ZFNs) has been used for total or partial deletion of lncRNAs in cells or to stop the expression of lncRNAs by insertion of polyadenylation signals between the promoter and the lncRNA sequence [214,217,218,219]. While the CRISPR-Cas system has been applied for complete knockdown and constitutive inhibition of lncRNAs, it is difficult to avoid unpredictable off-target effects using this strategy [220,221,222,223,224,225].

The CRISPR-Cas system can also be applied to achieve lncRNA overexpression by inserting a strong promoter upstream of the gene [130]. However, the most widely applied strategy for gain-of-function analysis is ectopic overexpression using transient transfection of an expression vector or the lncRNA itself transcribed in vitro with the 5′ cap and/or 3′ poly-adenylation or viral transduction [29,39,40]. This strategy may be effective when lncRNA exerts its function in trans.

### 6.4. Mouse Models and Knockout Models for LncRNA Study

In vitro cell-based experiments, such as soft-agar colony formation assays, have been used to show that some lncRNAs are associated with cellular processes related to cell transformation [9,226,227]. Various models have been developed to clarify whether these lncRNAs exert similar functions in vivo. Human cancer cells have tumorigenicity, which is the ability of cancer cells to generate tumors in immunologically nonresponsive animals, including nude and NOD/SCID mice [227,228]. Injection of cancer cells with modulated expression of specific lncRNAs has enabled the identification of lncRNAs that contribute to tumorigenesis [29,39,40,100,229,230]. For example, knockdown of *UPAT* or *ASBEL* reduces the tumorigenicity of colon or ovarian cancer cells in mice [29,39,40].

Only a few studies thus far have reported lncRNA knockout mice with critical developmental phenotypes, including mice with knockout for *Fendrr*, *Linc-Pint* or *Pantr2* [200,201,202]. One limitation in creating knockout mouse models to examine the function of a specific lncRNA is that the lncRNA of interest is not always highly conserved in humans and mice or other vertebrates [231]. Nevertheless, some human lncRNAs show partial conservation with other mammals such as mouse and lower vertebrates [11,89,190,232], suggesting the possibility of conserved functions of lncRNAs and conserved sequences or structural elements in lncRNAs that may be elucidated in the knockout mouse.

## 7. Conclusions

Advances in the human transcriptome have improved our comprehensive understanding of gene regulation in cancer. The presence of thousands of lncRNAs that are involved in various cellular functions may have important implications for the development and maintenance of cancer, forcing us to revise our view of cancer, including its causal origins and diagnosis and treatment strategies. Many studies have established that lncRNAs play an important role in tumor development, tumor progression, tumor cell survival and tumorigenesis. Because some lncRNAs show aberrant expression in tumor cells, the lncRNAs are currently under evaluation as biomarkers or direct therapeutic targets in clinical trials. The field, however, is still in its infancy, and applying lncRNAs into the clinical stage will take time. Despite the fact that strategies specifically targeting lncRNAs have provided promising results in mouse models, lncRNA-based therapeutic approaches have not yet reached the clinical stage and a variety of challenges remain unresolved.

Despite the rapid increase in the number of lncRNAs, the functions of lncRNAs not only in cancer cells but in physiological conditions in normal cells are still far from fully understood. The biggest challenge in functional analysis of lncRNAs is the inability to predict their function from their sequence, unlike proteins, and identification of binding partner and structural elements of lncRNA that confer cellular functions will lead to the understanding of their function. Moreover, some lncRNAs localize to their target genes by direct RNA-DNA interactions. The implementation of methodologies to identify proteins that bind to specific lncRNAs as well as detection of genome-wide DNA binding sites of lncRNAs using mass spectrometry analysis and massive sequence technologies will help determine the functions of lncRNAs [29,40,195,196,197,198,199]. In addition, techniques from other fields such as live cell RNA imaging techniques, structural biology approaches and RNA proteomics will be critical.

LncRNAs are now recognized as a major new class of genes in biology. LncRNAs also represent promising targets for diagnostic and tailored therapeutic applications. The creation of technologies that allow easy quantification and monitoring of expression by non-invasive approaches, such as using blood, urine and saliva, greatly increases the potential for the use of lncRNAs in clinical practice. CRISPR-based systems, synthetic oligonucleotide antagonists and oligonucleotides for the knockdown of lncRNAs or inhibition of specific lncRNA-protein interactions provide new opportunities for exploring the clinical relevance of lncRNAs. The discovery of novel lncRNAs, the identification of their function and behavior in various cancer subtypes, and the development of strategies targeting novel lncRNAs for diagnosis and tailored therapeutics are very promising and represent one of the major strategies for cancer treatment in the near future.

## Abbreviations

*ASBEL*	Antisense ncRNA in the ANA/BTG3 locus
ASO	Antisense oligonucleotides
BARD1	BRCA1-associated RING domain protein 1
*CCAT*1	Colon cancer-associated transcripts 1
*CCAT*2	Colon cancer-associated transcripts 2
CHART	Capture hybridization analysis of RNA targets
ChIRP	Chromatin isolation by RNA purification
CLC	Cancer LncRNA Census
EMT	Epithelial to mesenchymal transition
ENCODE	Encyclopedia of DNA Elements
*GAS*5	Growth arrest-specific transcript 5
*HOTAIR*	HOX antisense intergenic RNA
*LED*	LncRNA activator of enhancer domains
lncRNAs	Long ncRNAs
*lncRNA-ATB*	LncRNA-activated by TGF-β
ncRNAs	Noncoding RNAs
*NORAD*	Noncoding RNA activated by DNA damage
*MALAT*1	Metastasis associated in lung adenocarcinoma transcript
*MINCR*	MYC-induced long non-coding RNA
*NEAT*1	Nuclear enriched abundant transcript 1
*PANDA*	P21 associated ncRNA DNA damage activated
PCAWG	Pan-Cancer Analysis of Whole Genomes
*PCA*3	Prostate cancer antigen 3
*PCAT*1	Prostate cancer associated transcript 1
*PCGEM*1	Prostate-specific transcript 1
*PTENP*1	PTEN pseudogene
*PVT*1	Plasmacytoma variant translocation 1
Pol II	RNA polymerase II
RAP	RNA antisense purification
RIP	RNA immunoprecipitation
RNAi	RNA interference
RNP	Ribonucleoprotein
rRNAs	Ribosomal RNAs
*SAMMSON*	Survival associated mitochondrial melanoma specific oncogenic non-coding RNA
*SChLAP*1	Second chromosome locus associated with prostate-1
snRNAs	Small nuclear RNAs
snoRNAs	Small nucleolar RNAs
*TARID*	TCF21 antisense RNA inducing demethylation
TCGA	The Cancer Genome Atlas
*Terra*	Telomeric repeat-containing RNA
*TINCR*	Terminal differentiation-induced noncoding RNA
tRNAs	Transfer RNAs
*UPAT*	UHRF1 protein associated transcript
ZFNs	Zinc finger nucleases
